# Incremental prognostic value of coronary flow reserve determined by phase-contrast cine cardiovascular magnetic resonance of the coronary sinus in patients with diabetes mellitus

**DOI:** 10.1186/s12968-020-00667-3

**Published:** 2020-10-08

**Authors:** Shingo Kato, Kazuki Fukui, Sho Kodama, Mai Azuma, Tae Iwasawa, Kazuo Kimura, Kouichi Tamura, Daisuke Utsunomiya

**Affiliations:** 1 Department of Diagnostic Radiology, Yokohama City University Graduate School of Medicine, Yokohama, Japan; 2 Department of Cardiology, Kanagawa Cardiovascular and Respiratory Center, Yokohama, Japan; 3 Department Radiology, Kanagawa Cardiovascular and Respiratory Center, Yokohama, Japan; 4 Department of Cardiology, Yokohama City University Medical Center, Yokohama, Japan; 5 Department of Medical Science and Cardiorenal Medicine, Yokohama City University, Yokohama, Japan

**Keywords:** Diabetes mellitus, Coronary flow reserve, Magnetic resonance imaging, Phase contrast, Coronary sinus, Prognosis

## Abstract

**Background:**

Although non-invasive assessment of coronary flow reserve (CFR) by cardiovascular magnetic resonance (CMR) provides prognostic information for patients with diabetes mellitus (DM), the incremental prognostic value of CMR-derived CFR remains unclear.

**Purpose:**

To evaluate the incremental prognostic value of CMR-derived CFR for patients with DM who underwent stress CMR imaging.

**Materials and methods:**

A total of 309 patients with type 2 DM [69 ± 9 years; 244 (78%) male] assessed between 2009 and 2019 were retrospectively reviewed. Coronary sinus blood flow (CSBF) was measured using phase contrast (PC) cine CMR. CFR was calculated as the CSBF during adenosine triphosphate infusion divided by that at rest. Major adverse cardiac events (MACE) were defined as death, acute coronary syndrome, hospitalization due to heart failure exacerbation, or sustained ventricular tachycardia. The incremental prognostic value of CFR over clinical and CMR variables was assessed by calculating the C-index and net reclassification improvement (NRI).

**Results:**

During a median follow-up of 3.8 years, 42 patients (14%) experienced MACE. The annualized event rate was significantly higher among patients with CFR < 2.0, regardless of the presence of late gadolinium enhancement (LGE) (1.4% vs. 9.8%, *p* = 0.011 in the LGE (−) group; 1.8% vs. 16.9%, *p* < 0.001 in the LGE (+) group). In addition, this trend was maintained in the subgroups stratified by presence or absence of ischemia (0.3% vs. 6.7%, *p* = 0.007 in the ischemia (−) group; 3.9% vs. 17.1%, *p* = 0.001 in the ischemia (+) group). Adding CFR to the risk model (age + gender + left ventricular ejection fraction + %LGE + %ischemia) resulted in a significant increase of the C-index from 0.838 to 0.870 (*p* = 0.038) and an NRI of 0.201 (0.004–0.368, *p* = 0.012).

**Conclusion:**

PC cine CMR-derived CFR of the coronary sinus may be useful as a prognostic marker for DM patients, incremental to common clinical and CMR parameters. Due to the high prevalence of coronary microvascular dysfunction, the addition of CFR to conventional vasodilator stress CMR imaging may improve risk stratification for patients with DM.

## Introduction

Diabetes mellitus (DM) is a major risk factor for atherosclerotic diseases, and is associated with an approximately twofold increased risk of coronary artery disease (CAD), stroke, and cardiac mortality [1]. Early detection of high risk DM patients who are at risk of developing CAD is important to improve the clinical outcome. Previous studies has shown that the coronary flow reserve (CFR), an index of coronary vascular dysfunction, assessed by positron emission tomography (PET) is an independent prognostic factor in patients with DM [2]. PET-derived CFR could be useful as a non-invasive mean to risk stratify the DM patients, however, radiation exposure is a non-negligible limitation for CFR measurement using PET.

Phase-contrast (PC) cine cardiovascular magnetic resonance (CMR) of the coronary sinus has emerged as a non-invasive means of quantifying global coronary flow reserve (CFR) without radiation exposure [3–5]. Validation studies have been performed using phantom models [6], animal experimental model using flow probes [7] and PET [3]. Recent studies have demonstrated the prognostic value of CMR-derived CFR for patients with known or suspected CAD [8, 9]. In patients with DM, the prevalence of impaired CFR (< 2.0) was significantly higher compared with that in those without DM, and impairment of CFR was a significant predictor for adverse cardiac events [10]. To date, the incremental prognostic value of CFR over conventional CMR variables remains unclear. Therefore, the aim of this study was to evaluate the incremental prognostic value of CFR in known or suspected CAD patients with DM who underwent stress CMR.

## Materials and methods

### Study population

**Fig. 1:**
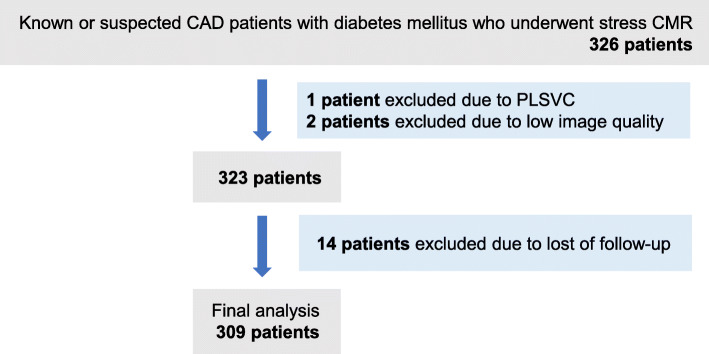
Flow chart of patient selection. CAD, coronary artery disease; CMR, cardiovascular magnetic resonance; PLSVC, persistent left superior vena cava

This retrospective observational study included 326 type 2 DM patients with known or suspected CAD. Known CAD was defined as evidence of myocardial infarction, previous percutaneous coronary intervention or coronary artery bypass graft, or angiographically significant coronary stenosis (> 70% stenosis in any epicardial coronary artery or > 50% of the left main coronary artery). Suspected CAD was defined as having symptoms suspicious of myocardial ischemia (chest pain, dyspnea on exertion etc.) or ischemic changes on electrocardiogram (ECG; ST segment depression, inverted T waves etc.). The diagnosis of DM was made according to the Japanese Clinical Practice Guidelines for Diabetes. Figure 1 shows the flow chart of patient selection. All patients underwent stress CMR to evaluate myocardial ischemia between 2009 and 2019. One patient was excluded due to having a persistent left vena cava. Two patients were excluded due to low image quality. Follow-up information was obtained from 96% of the population. Finally, 309 patients were included as the study cohort. This study was approved by the institutional review board, and written informed consent was waived because of the retrospective design.

### Acquisition of CMR images

**Fig. 2:**
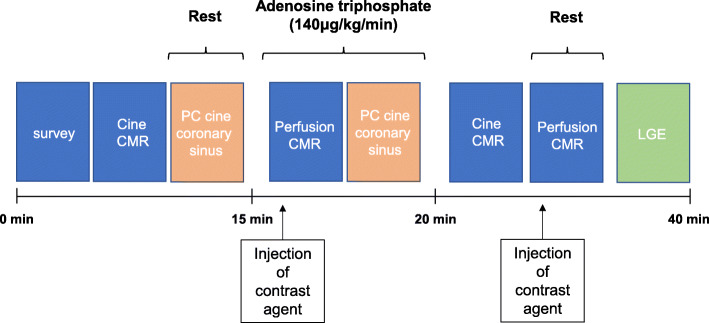
Cardiovascular magnetic resonance protocol. CMR, cardiovascular magnetic resonance; PC, phase-contrast; LGE, late gadolinium enhancement;

CMR images were acquired using a 1.5T CMR scanner (Achieva; Philips Healthcare, Best, The Netherlands) equipped with a 32-channel cardiac coil. Our CMR protocol included cine CMR, PC cine CMR of the coronary sinus, stress perfusion CMR, and late gadolinium enhancement (LGE) MRI (Fig. 2). We visualized ventricular size and function on cine images acquired under the following conditions: cine balanced steady-state free-precession (repetition intervals, 4.1 ms; echo intervals, 1.7 ms; flip angle, 55°; field of view, 350 × 350 mm; acquisition matrix, 128 × 128; slice thickness, 10 mm; number of phases per cardiac cycle, 20). We evaluated the presence and severity of myocardial ischemia using first-pass perfusion CMR images acquired under the following conditions: turbo field echo sequence (4 short-axis slices/2 RR intervals; repetition time, 2.9 msec; echo time, 1.4 msec; flip angle, 40°; saturation delay, 200 msec; field of view, 360 × 324 mm; acquisition matrix, 192 × 172; reconstruction matrix, 256 × 230; slice thickness, 8 mm). Immediately after the sequence for perfusion CMR started, gadolinium contrast (gadopentetate dimeglumine, Magnevist; Bayer, Berlin, Germany; or gadoterate meglumine, Magnescope; Guerbet, Paris, France) was injected into the right antecubital vein at a dose of 0.05 mmol/kg and a flow rate of 4 mL/s. Pharmacological stress was induced by continuous injection of adenosine triphosphate (ATP; 140 μg/kg/min) into the left antecubital vein. The interval between stress and resting perfusion CMR image acquisition was at least 10 min. We investigated the presence and degree of myocardial infarction or scarring by acquiring LGE images in the same planes as the cine images using inversion recovery-prepared gradient-echo sequences under the following conditions: repetition duration, 4.3 ms; echo duration, 1.3 ms; flip angle, 15°; field of view, 380 × 380 mm; acquisition matrix, 256 × 180; slice thickness, 10 mm). All patients refrained from consuming caffeinated beverages for at least 24 h before the CMR.

### Acquisition of PC cine CMR of the coronary sinus

**Fig. 3:**
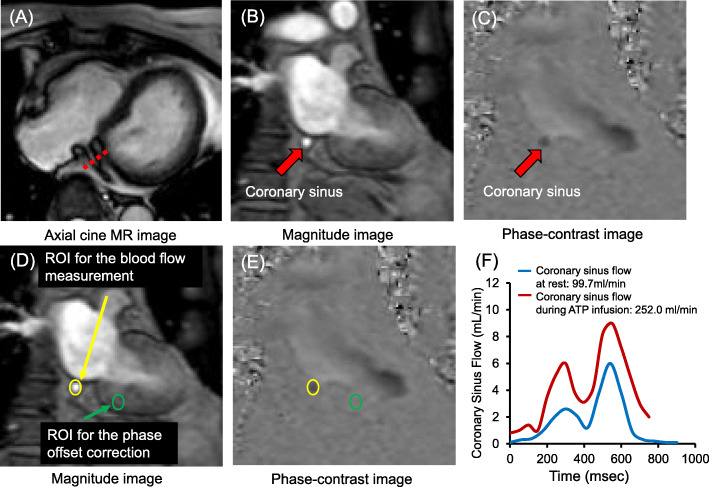
Slice selection and location of ROI for flow measurements in the coronary sinus. **a-c** Slice selection for acquisition of phase-contrast cine images of the coronary sinus. **d, e** Location of region of interest (ROI) for blood flow measurements and phase-offset correction. **f** Representative blood flow in the coronary sinus. Coronary sinus blood flow (CSBF) typically peaks twice during the systolic and diastolic phases. ROI, region of interest; ATP, adenosine triphosphate

Figure 3 shows the position of the coronary sinus, analysis of coronary sinus blood flow (CSBF), and blood flow curves of the coronary sinus on CMR. The imaging plane for measuring blood flow was positioned perpendicular to the coronary sinus at 1.5–2.0 cm from its ostium on axial cine CMR images (Fig. 3a). We acquired PC cine CMR of the coronary sinus while the patients held their breath (repetition duration, 7.3 ms; echo duration, 4.4 ms; flip angle, 10°; field of view, 380 × 228 mm; acquisition matrix, 160 × 160; reconstruction matrix, 256 × 256; reconstruction resolution, 1.48 × 1.48 mm; number of phases per cardiac cycle, 20; velocity encoding, 50 cm/s; slice thickness, 6 mm) (Fig. 3b, c). Time of breath-hold for PC cine CMR of the coronary sinus is approximately 15–20 s.

### CMR image analysis

We analyzed cine, perfusion, PC cine, and LGE images using an Extend MR WorkSpace workstation (Philips Healthcare). Manual tracing of epicardial and endocardial borders of the left ventricle (LV) on short-axis cine images was performed, while excluding the papillary muscles, to measure LV volume, mass, and ejection fraction (LVEF). LV mass was calculated as the sum of the myocardial volume areas multiplied by the specific gravity (1.05 g/mL) of myocardial tissue [11]. The contours of the coronary sinus were manually traced to quantify CSBF, and velocity in the adjacent myocardium was measured to perform phase-offset correction (Fig. 3d, e) by subtracting the background velocity of the adjacent myocardium from the velocity of the CSBF at each acquired phase. For phase-offset correction, we drew the region-of-interest on myocardium separately for each cardiac phase. CSBF was calculated by integrating the product of the cross-sectional area and mean velocity in the coronary sinus (Fig. 3F).

We calculated the following:

- ΔCSBF (mL/min) = CSBF during ATP infusion (mL/min) – CSBF at rest (mL/min).
- CFR = CSBF during ATP infusion (mL/min) / CSBF at rest (mL/min).

For the analysis of perfusion CMR, We chose 3 from 4 short axis slices in order from most apical slice (apical-, mid- and basal slices). The ischemic segment was defined as a myocardium that appears hypointense after peak myocardial enhancement along the coronary artery territory on perfusion CMR, located within viable myocardium (unenhanced myocardium on LGE CMR). We calculated % ischemia by dividing the LV myocardium into 32 subsegments (endocardial and epicardial sectors for each of the American Heart Association 16-segment models). Each subsegment represents 3% of the total LV myocardium. For example, if patient had ischemia in 3 myocardial subsegments, ischemic extent was calculated as 9%. The criterion for categorization as high risk was > 10% ischemia, in accordance with the nuclear sub-study of the COURAGE trial [12]. LGE images were quantitatively evaluated using the manual planimetry method and the %LGE was calculated.

### Follow-up of adverse events

Prognostic information was obtained from the electronic medical records. Major adverse cardiovascular events (MACE) were defined as cardiovascular death, non-cardiovascular death, acute coronary syndrome, unstable angina, hospitalization for heart failure, and sustained ventricular tachyarrhythmia. The first event after CMR assessment was recorded. Time to event was calculated as time from the CMR scan to the first event. Patients who did not experience MACE were censored at the time of the last-follow-up. Adverse events were investigated under blinding to all CMR findings.

### Statistical analysis

Data were statistically analyzed using SPSS (version 17.0, Statistical Package for the Social Sciences, International Business Machines, Inc., Armonk, New York, USA, MedCalc for Windows (version 14.8.1, MedCalc Software, Ostend, Belgium), and R (version 3.6.3, The R Foundation for Statistical Computing, Vienna, Austria). Continuous values were presented as means ± standard deviation and categorical values were presented as number (%). Normality was determined using Shapiro-Wilk tests. Normally distributed values were compared using unpaired t tests, and non-normally distributed values were compared using Mann-Whitney U tests. The significance of differences in categorical variables was calculated using the chi-square test. Multivariable associations with MACE were determined by Cox proportional hazards regression, and event-free survival stratified by CFR < 2.0 was estimated from Kaplan-Meier survival curves. Two models of multivariable Cox regression were performed. In model 1, continuous variables were used for LGE, ischemia, and CFR, while in model 2, categorical variables were used. Multivariable logistic regression analysis was performed to combine clinical and imaging parameters using following formula: 0.298 + 1.008 × age (years) + 0.49 × gender (male) + 0.967 × LVEF (%) + 1.010 × %LGE + 1.166 × %ischemia. The incremental prognostic value of CFR over this multivariable logistic regression model was assessed by comparing the C-index before and after adding CFR (DeLong’s test) and calculating the net reclassification improvement (NRI). Intra- and interobserver reliability of CSBF measurement was assessed in 10 patients by calculating intra-class correlation coefficients (ICC). A *p*-value < 0.05 was considered statistically significant.

## Results

### Patients’ characteristics

Table 1 summarizes the characteristics of the entire cohort. The mean age of DM patients was 69 ± 9 years (median: 70 years; range: 37–88). Seventy-eight percent of patients were male. The mean HbA1c was 6.8 ± 0.9%. The mean LVEF was 58 ± 12%, and LGE was identified in 46% of patients. Ischemia was inducible in 41% of patients. Mean global CFR was 2.7 ± 0.9. The prevalence of impaired global CFR (< 2.0) was 17%. The primary indications for stress CMR were chest pain (69%), dyspnea (18%), and ECG abnormalities (7%). Compared with patients without MACE, those with MACE had a higher prevalence of dyslipidemia, and higher rates of statin and nitroglycerin treatment (*p* < 0.05). Regarding CMR variables, patients with MACE had decreased LVEF, and a higher prevalence of LGE and ischemia (Table 1).

**Table 1:** Patient characteristics

	All patients ***N*** = 309	Patients without MACE ***N*** = 267	Patients with MACE ***N*** = 42	*P-value
**Clinical variables**				
**Age, years**	69 ± 9	69 ± 9	70 ± 9	0.45
**Male**	244 (78%)	212 (79%)	32 (76%)	0.79
**BMI, kg/m**^**2**^	25 ± 4	25 ± 3	25 ± 4	0.87
**Hypertension**	193 (62%)	163 (61%)	30 (71%)	0.26
**Dyslipidemia**	180 (58%)	148 (55%)	32 (76%)	0.017
**Smoking**	31 (10%)	25 (9%)	6 (14%)	0.47
**History of CAD**	176 (56%)	147 (55%)	29 (69%)	0.12
**Blood tests**				
**HbA1c, %**	6.8 ± 0.9	6.8 ± 0.9	6.8 ± 0.9	0.97
**LDL cholesterol**	99 ± 29	98 ± 29	104 ± 30	0.21
**eGFR**	67 ± 16	68 ± 15	62 ± 17	0.048
**Medications**				
**Aspirin**	183 (59%)	152 (57%)	31 (74%)	0.057
**Beta-blocker**	124 (40%)	105 (39%)	19 (45%)	0.58
**Statin**	171 (55%)	139 (52%)	32 (76%)	0.006
**Calcium channel blocker**	116 (37%)	100 (37%)	16 (38%)	0.92
**Nitroglycerin**	101 (33%)	81 (30%)	20 (48%)	0.041
**ACE inhibitor/ARB**	124 (38%)	104 (39%)	20 (47%)	0.37
**Oral hypoglycemic agent**	220 (71%)	190 (71%)	30 (71%)	0.89
**Insulin**	21 (7%)	16 (6%)	5 (12%)	0.27
**CMR variables**				
**LVEDV, mL**	129 ± 46	128 ± 41	137 ± 67	0.23
**LVESV, mL**	57 ± 36	55 ± 34	69 ± 49	0.022
**LV mass, g**	96 ± 25	94 ± 26	102 ± 24	0.063
**LVEF, %**	58 ± 12	59 ± 12	53 ± 13	0.003
**Presence of LGE**	143 (46%)	115 (43%)	28 (67%)	0.007
**Presence of ischemia**	124 (40%)	87 (33%)	37 (88%)	< 0.001

Data are expressed as mean ± standard deviation or number (%)

*P-value represents significance of difference between patients with MACE and those without

*ACE* angiotensin converting enzyme, *ARB* angiotensin receptor blocker, *BMI* body mass index, *CAD* coronary artery disease, *CFR* coronary flow reserve, *CMR* cardiac magnetic resonance, *eGFR* estimated glomerular filtration rate, *LDL* low-density lipoprotein, *LGE* late gadolinium enhancement, *LV* left ventricular, *LVEDV* left ventricular end-diastolic volume, *LVEF* left ventricular ejection fraction, *LVESV* left ventricular end-systolic volume, MACE major adverse cardiac events

### Comparison of CSBF and CFR between patients with and without adverse events

Table 2 compares CSBF and CFR between patients with and without MACE. Across the overall cohort, the baseline CSBF was 91.8 ± 37.6 mL/min and CSBF during ATP infusion was 234.4 ± 81.1 mL/min, resulting in a CFR of 2.7 ± 0.9. Baseline CSBF was significantly higher (121.2 ± 47.9 mL/min vs. 87.2 ± 33.5 mL/min, *p* < 0.001), **Δ**CSBF was significantly lower (116.5 ± 52.6 mL/min vs. 143.1 ± 67.7 mL/min, *p* < 0.001), and CFR was significantly lower (2.1 ± 0.4 vs. 2.8 ± 0.9, *p* < 0.001) in patients with MACE compared to those without. Prevalence of CFR < 2.0 was significantly higher in patients with MACE compared to those without (60% vs. 11%, *p* < 0.001).

**Table 2:** Comparison of coronary sinus blood flow and coronary flow reserve

	All patients ***N*** = 309	Patients without MACE ***N*** = 267	Patients with MACE ***N*** = 42	*P-value
**CSBF at rest (mL/min)**	91.8 ± 37.6	87.2 ± 33.5	121.2 ± 47.9	< 0.001
**CSBF during ATP infusion (mL/min)**	234.4 ± 81.1**	230.3 ± 80.8**	237.8 ± 83.9**	0.58
**ΔCSBF (mL/min)**	139.5 ± 66.5	143.1 ± 67.7	116.5 ± 52.6	0.016
**Coronary flow reserve**	2.7 ± 0.9	2.8 ± 0.9	2.0 ± 0.4	< 0.001
**Coronary flow reserve < 2.0**	55 (17%)	30 (11%)	25 (60%)	< 0.001

Data are expressed as mean ± standard deviation or number (%)

* *P*-values represent the difference between patients with and without MACE. ** *P* < 0.05 vs. CSBF at rest

ΔCSBF = CSBF during ATP infusion – CSBF at rest

Coronary flow reserve = CSBF during ATP infusion / CSBF at rest × 100

*ATP* adenosine triphosphate, *CSBF* coronary sinus blood flow, *MACE* major adverse cardiac events

### Prognostic value of global CFR in DM patients

**Fig. 4:**
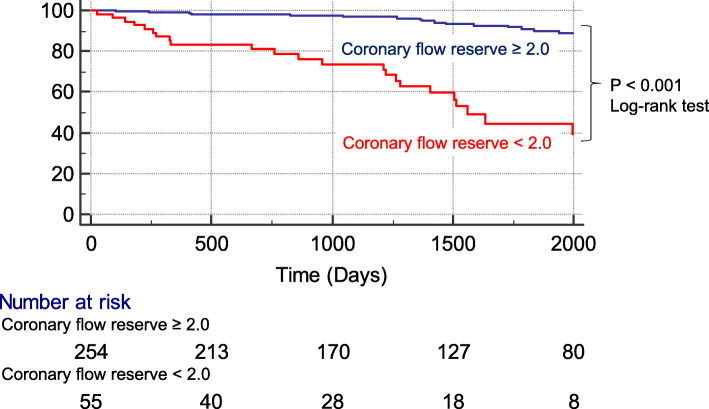
Kaplan-Meier event-free survival curves for patients with major adverse cardiac events

**Fig. 5:**
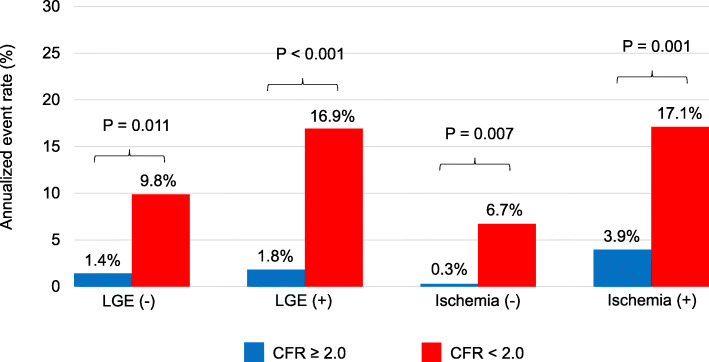
Annualized adverse event rates stratified by CFR according to the presence or absence of LGE or ischemia. The annualized rates of major adverse cardiac events were significantly higher in patients with impaired CFR (< 2.0), irrespective of LGE or ischemia. CFR, coronary flow reserve; LGE, late gadolinium enhancement

Forty-two (14%) patients experienced MACE over a median follow-up period of 3.8 years (cardiovascular death, *n* = 10; non-cardiovascular death, *n* = 6; acute coronary syndrome, *n* = 15; hospitalization for heart failure, n = 10; sustained ventricular tachyarrhythmia, *n* = 1). Kaplan-Meier event-free survival curves for adverse events in patients with and without DM stratified by a CFR cutoff of 2.0. The rates of MACE were significantly higher in patients with CFR < 2.0 (*p* < 0.001) (Fig. 4). Figure 5 shows the annualized event rates stratified by global CFR in the presence or absence of LGE and ischemia. The annualized event rate was significantly higher among patients with CFR < 2.0, regardless of the presence of LGE (1.4% vs. 9.8%, *p* = 0.011 in the LGE (−) group; 1.8% vs. 16.9%, *p* < 0.001 in the LGE (+) group). In addition, this trend was maintained in the subgroups stratified by presence or absence of ischemia (0.3% vs. 6.7%, *p* = 0.007 in the ischemia (−) group; 3.9% vs. 17.1%, *p* = 0.001 in the ischemia (+) group) (Fig. 5).

### Multivariable analysis and incremental prognostic value

**Fig. 6:**
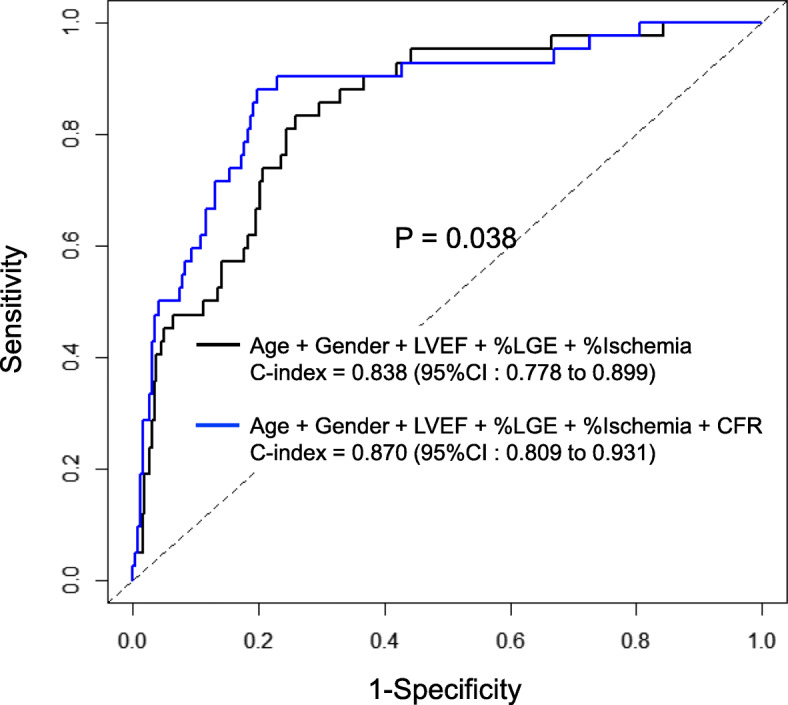
Comparison of receiver operating characteristics curves for predicting major adverse cardiac events. CFR, coronary flow reserve; LGE, late gadolinium enhancement; LVEF, left ventricular ejection fraction

After adjustment for clinical and imaging risk factors, ischemia on perfusion CMR [hazard ratio: 1.11; 95% confidence interval: 1.07–1.16, *p* < 0.001)] and CFR (hazard ratio: 0.34; 95% confidence interval: 0.19–0.61, *p* < 0.001) were identified as significant predictors of MACE. After conversion of LGE, ischemia, and CFR into categorical variables, the multivariable model revealed that ischemia > 10% (hazard ratio: 2.45; 95% confidence interval: 1.24–4.85, *p* = 0.009) and CFR < 2.0 (hazard ratio: 3.36; 95% confidence interval: 3.05–13.29, *p* < 0.001) were independent predictors for MACE (Table 3). Figure 6 shows the receiver operating characteristic curves for predicting MACE. The C-index for the combination of age, gender, LVEF, % LGE and % ischemia was 0.838 (95% confidence interval: 0.778–0.899). Adding CFR to this model resulted in a significant increase in the C-index from 0.838 to 0.870 (*p* = 0.038) and an NRI of 0.201 (0.004–0.368, *p* = 0.012).

**Table 3:** Multivariable Cox regression analysis of predictors of MACE

	All patients ***N*** = 309	Patients without MACE ***N*** = 267	Patients with MACE ***N*** = 42	*P-value
**CSBF at rest (mL/min)**	92 ± 38	87 ± 34	121 ± 48	< 0.001
**CSBF during ATP infusion (mL/min)**	234 ± 81**	230 ± 81**	238 ± 84**	0.58
**ΔCSBF (mL/min)**	140 ± 67	143 ± 68	117 ± 53	0.016
**Coronary flow reserve**	2.7 ± 0.9	2.8 ± 0.9	2.0 ± 0.4	< 0.001
**Coronary flow reserve < 2.0**	55 (17%)	30 (11%)	25 (60%)	< 0.001

Data are expressed as mean ± standard deviation or number (%)

* *P*-values represent the difference between patients with and without MACE. ** *P* < 0.05 vs. CSBF at rest

ΔCSBF = CSBF during ATP infusion – CSBF at rest. Coronary flow reserve = CSBF during ATP infusion / CSBF at rest × 100

*ATP* adenosine triphosphate, *CSBF* coronary sinus blood flow, *MACE* major adverse cardiac events

### Intra- and inter-observer reproducibility

Regarding intra-observer reproducibility, the ICC was 0.96 (95% confidence interval: 0.94–0.99) for rest CSBF and 0.90 (95% confidence interval: 0.86–0.94) for stress CSBF measurements. In terms of inter-observer reproducibility, the ICC was 0.90 (95% confidence interval: 0.85–0.95) for rest CSBF and 0.91 (95% confidence interval: 0.87–0.95) for stress CSBF measurements.

## Discussion

This study showed that CFR provides incremental prognostic information over standard clinical and CMR risk factors, including LVEF, LGE, and ischemia. In addition, the annualized event rate was significantly higher among patients with CFR < 2.0, regardless of the presence of LGE and ischemia, suggesting that CMR-derived CFR could be useful for risk stratification even in patients without overt disease. Due to the high prevalence of coronary microvascular dysfunction, addition of CFR to conventional stress CMR imaging may be useful for better risk stratification for patients with DM.

DM is associated with an increased risk of various atherosclerotic diseases, such as multivessel CAD [13, 14], diffuse but non-obstructive atherosclerosis [13, 15], and microvascular dysfunction [16]. Several complex mechanisms link DM to these vascular abnormalities, including hyperglycemia and insulin resistance [16], systemic inflammation, and autonomic dysfunction [17]. The prevalence of microvascular dysfunction is high in patients with DM. A ^82^Rb PET/computed tomography study of patients with type 2 DM and no overt cardiovascular disease found reduced CFR (< 2.5) in 16.7% of controls, and 40.0 and 83.3% of DM patients without and with albuminuria, respectively [8]. Another study using ^13^N-ammonia PET showed significantly reduced endothelium-dependent (cold pressor test) and independent (adenosine-induced hyperemia) coronary vasodilator function in patients with DM types 1 and 2 [16]. Microvascular dysfunction is associated with poor clinical outcome in patients with DM. Doppler-derived coronary flow velocity assessment of the left anterior descending artery demonstrated a high annualized event rate (death, non-fatal myocardial infarction) of 13.9% in type 2 DM patients with reduced coronary flow velocity reserve, and 2.0% in those with preserved coronary velocity reserve [2]. Therefore, accurate assessment of microvascular function is important for the clinical management of patients with DM.

CMR has been recognized as a useful imaging modality for identifying subclinical myocardial abnormalities in patients with DM. Owing to its higher spatial resolution than single photon emission computed tomography SPECT [18], unrecognized myocardial infarction is detectable by LGE CMR, and is closely associated with future MACE among patients with DM [19]. Stress perfusion CMR enables quantitative assessment of myocardial ischemia, and effectively reclassifies the risk of MACE in patients with DM [20]. PC cine CMR of the coronary sinus has emerged as a CMR method to evaluate global CFR, and its prognostic value of in patients with known or suspected CAD has been demonstrated [8, 9]. Adding CFR to stress perfusion CMR resulted in improved accuracy in detecting multivessel CAD [21]. In a recent study, DM patients had higher rates of CMR-derived CFR impairment (< 2.0) compared with those without DM (17% vs. 7%, *p* < 0.001), and patients with impaired CFR had a substantially higher annual risk of adverse events [10]. However, the incremental prognostic value of CMR-derived CFR over conventional CMR parameters, such as LVEF, LGE, and ischemia, had not been investigated. Our study showed that CMR-derived CSBF CFR provides prognostic information independent of clinical and CMR variables, such as age, gender, LVEF, and ischemia. Moreover, addition of CFR in this model resulted in significant improvement of the C-index from 0.838 to 0.870 and an NRI of 0.201. In our study, the CFR cut-off value of 2.0 was used for the definition of CFR impairment, which was derived from the previous PET studies which investigated the prognostic value of CFR in patients with CAD [22–24]. These results suggested a new role for CMR-derived CFR in identifying high risk DM patients beyond conventional CMR parameters. In addition, previous PET studies have shown that CFR is blunted in patients with hypertension [25], metabolic syndrome [26], smoking [27], dyslipidemia [28] and chronic kidney disease [29], even without obstructive CAD. Therefore, CMR-derived CFR may also detect the impairment of CFR, and may be able to predict worse clinical outcome in these conditions. Furthermore, coronary endothelium-dependent vasodilator response can be assessed by measuring the change of CSBF to cold pressor test using PC cine CMR. Previous studies have shown the feasibility of this technique in healthy subjects [30], asymptomatic women with cardiovascular risk factors [31] and young smokers [32]. Further study is necessary to clarify clinical value of this technique.

Another important finding is that resting CSBF was significantly higher, but CFR was significantly lower, in patients with adverse events (Table 2). One explanation for this phenomenon is that CSBF is already elevated at rest to account for the ischemia caused by epicardial coronary stenosis, microvascular dysfunction, or both, whereas the reserve capacity for pharmacological stress is decreased in patients with MACE. A similar observation was reported in the PET literature [33]. The clinical importance of resting indices for the assessment of physiological ischemia, such as the instantaneous wave-free ratio, has been recently recognized [34, 35]. The instantaneous wave-free ratio has been shown to be non-inferior to fractional flow reserve in guiding percutaneous coronary intervention. Further study is required to assess whether PC CMR-derived resting CSBF can predict MACE.

### Study limitations

There are several limitations to our study. First, this was a retrospective, single-center, observational study involving a limited number of patients. Therefore, a prospective, multicenter study is desirable to generalize our observations. Second, the exact mechanisms through which non-invasive CFR measures are related to higher cardiac mortality could not be determined. Third, lower temporal resolution of our perfusion CMR technique may decrease the detectability of myocardial ischemia (4 short axis slices over 2 RR intervals). Forth, although CFR measurements can evaluate the global CFR, they cannot determine which particular coronary territory regions exhibit reduced CFR. In addition, CFR derived by CSBF is not necessarily specific to the coronary pathophysiology, that is, epicardial CAD, microcirculatory disease by systemic diseases, various cardiomyopathies, or any of these combinations can affect CFR. Fifth, additional scan time of PC cine CMR of the coronary sinus over the conventional stress CMR protocol, and analysis time of blood flow measurement and phase-offset correction are practical limitations of this method. Recently, quantitative analysis of stress perfusion CMR has become more feasible. A recent study demonstrated that the automated pixel-wise quantitative myocardial perfusion mapping by CMR can detect obstructive CAD and coronary microvascular dysfunction [36]. New techniques of quantitative stress perfusion CMR may overcome the limitation of the CFR measurement by PC CMR.

## Conclusions

PC cine CMR-derived CFR of the coronary sinus may be useful as a prognostic marker for DM patients, incremental to common clinical and CMR parameters. Due to the high prevalence of coronary microvascular dysfunction, addition of CFR to conventional stress CMR imaging may improve risk stratification for patients with DM.

## Data Availability

The datasets during and/or analyzed during the current study available from the corresponding author on reasonable request.

## Abbreviations

ATP: Adenosine triphosphate
CAD: Coronary artery disease
CFR: Coronary flow reserve
CMR: Cardiovascular magnetic resonance
CSBF: Coronary sinus blood flow
DM: Diabetes mellitus
ECG: Electrocardiogram
LGE: Late gadolinium enhancement
LV: Left ventricle/left ventricular
LVEF: Left ventricular ejection fraction
MACE: Major adverse cardiovascular events
NRI: Net reclassification index
PC: Phase-contrast
PET: Positron emission tomography
PLSVC: Persistent left superior vena cava
SPECT: Single photon emission computed tomography
